# Case Report: Cytoreductive nephrectomy and thrombectomy in metastatic renal cell carcinoma with venous tumor thrombus: a case using indocyanine green fluorescence-guided laparoscopy

**DOI:** 10.3389/fonc.2025.1484844

**Published:** 2025-06-04

**Authors:** Xiaoyong Dong, Jie Zhang, Yongjun Pan, Tiejun Xie, Jinhua Wang

**Affiliations:** Department of Urology, The Beibei Affiliated Hospital of Chongqing Medical University, The Ninth People’s Hospital of Chongqing, Chongqing, China

**Keywords:** fluorescence-guided laparoscopy, renal cell carcinoma, tumor thrombus, cytoreductive nephrectomy, indocyanine green

## Abstract

**Background and Objective:**

Indocyanine green (ICG) is a widely utilized non-radiative fluorescent contrast agent that has progressively demonstrated unique advantages in urological endoscopic surgeries in recent years. Despite its potential advantages, to date, no reports have detailed the use of fluorescence laparoscopy in cytoreductive nephrectomy and thrombectomy(CNT) for metastatic renal cell carcinoma (mRCC) complicated by renal vein tumor thrombus. This paper presents a single-case clinical experience of CNT for mRCC with renal vein tumor thrombus, with procedures guided by ICG fluorescence-guided laparoscopy.

**Methods:**

We retrospectively analyzed the clinical data of a patient diagnosed with mRCC complicated by renal vein tumor thrombus. The patient underwent CNT at Beibei Hospital, Chongqing Medical University on November 7, 2023.

**Results:**

A 65-year-old male had a left renal tumor, systemic metastases, and left renal vein tumor thrombus shown by preoperative computed tomography(CT). Before surgery, he received seven cycles of anti PD-1 (Toripalimab) immunotherapy and 20 days of pazopanib (anti-vascular TKI), which was stopped due to liver impairment. After treatment, the left renal lesion shrank and some metastases vanished. As the patient was in good condition and willing to have surgery, CNT was done under ICG fluorescence-guided laparoscopy successfully. Post-operative pathology confirmed left kidney clear cell carcinoma with left renal vein tumor thrombus. No complications occurred, and the patient recovered and was discharged. Regular rechecks of 16 months showed no tumor progression.

**Conclusion:**

ICG fluorescence real-time imaging technology is characterized by real-time dynamics, convenience and safety. Performing CNT for mRCC with tumor thrombus in the renal vein under ICG fluorescence navigation laparoscopy is safe and feasible.

## 1 Introduction

Renal cell carcinoma(RCC) is one of the common malignant tumors in the urinary system, and its incidence accounts for 3%-5% of adult malignant tumors (1). Involvement of the renal vein is one of the biological characteristics of RCC. The incidence of RCC with associated venous tumor thrombus has been reported to be 4%-10% (2, 3). Currently, radical nephrectomy combined with removal of the venous tumor thrombus is considered the first choice for the treatment of renal cell carcinoma with renal vein tumor thrombus (4). The difficulty during the operation is to determine the location of the renal vein tumor thrombus during the operation and how to avoid the detachment of the tumor thrombus.

Accurate surgical operations pursue “efficiency and safety”, which requires strict preoperative assessment, surgical planning, surgical implementation and perioperative management. The invasiveness of the surgery requires surgeons to balance the relationship between clearing the lesion, protecting the organs and controlling the injury. Intraoperative visualization tools are important auxiliary means. Indocyanine green (ICG) is a widely used non-radiative fluorescent contrast agent and has gradually shown unique advantages in urological endoscopic surgery in recent years (5). Our department successfully performed a case of cytoreductive nephrectomy for metastatic renal cell carcinoma(mRCC) with renal vein tumor thrombus under ICG fluorescence laparoscopy on November 7, 2023, and is hereby reported.

## 2 Case report

**Figure 1:**
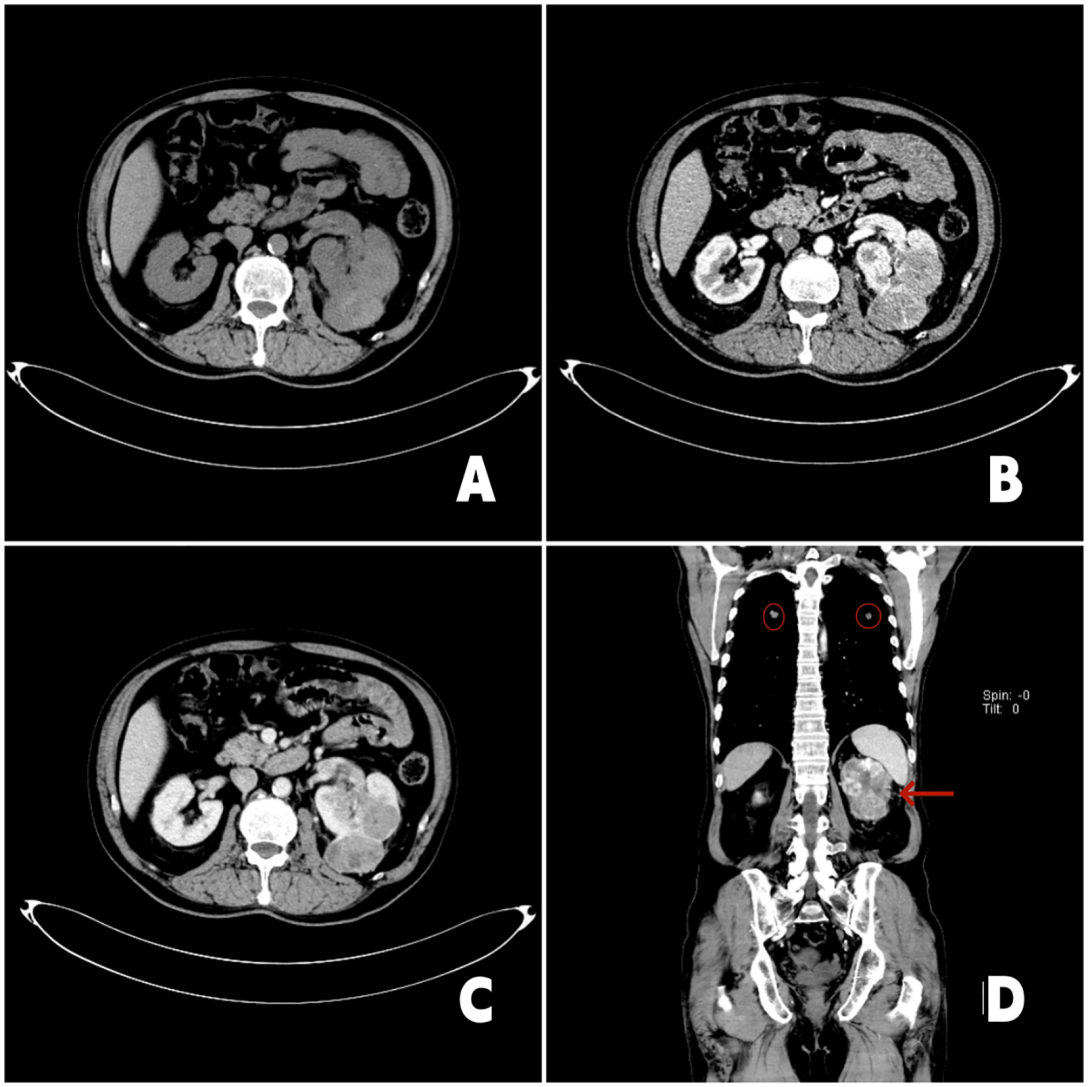
2023-3-11 chest enhanced CT and urinary CT: **(A)** Axial CT scan image showed a irregular mass shadow in the left kidney; **(B)** The image in the arterial phase of enhanced CT showed irregular enhancement of the left renal mass; **(C)** The enhancement pattern is characterized by rapid wash-in and wash-out. The left renal vein is thickened, and soft tissue shadow is seen inside; **(D)** Coronal enhanced CT image. The red arrow in the image points to the left renal tumor, and the red circle points to the lung metastasis.

**Figure 2:**
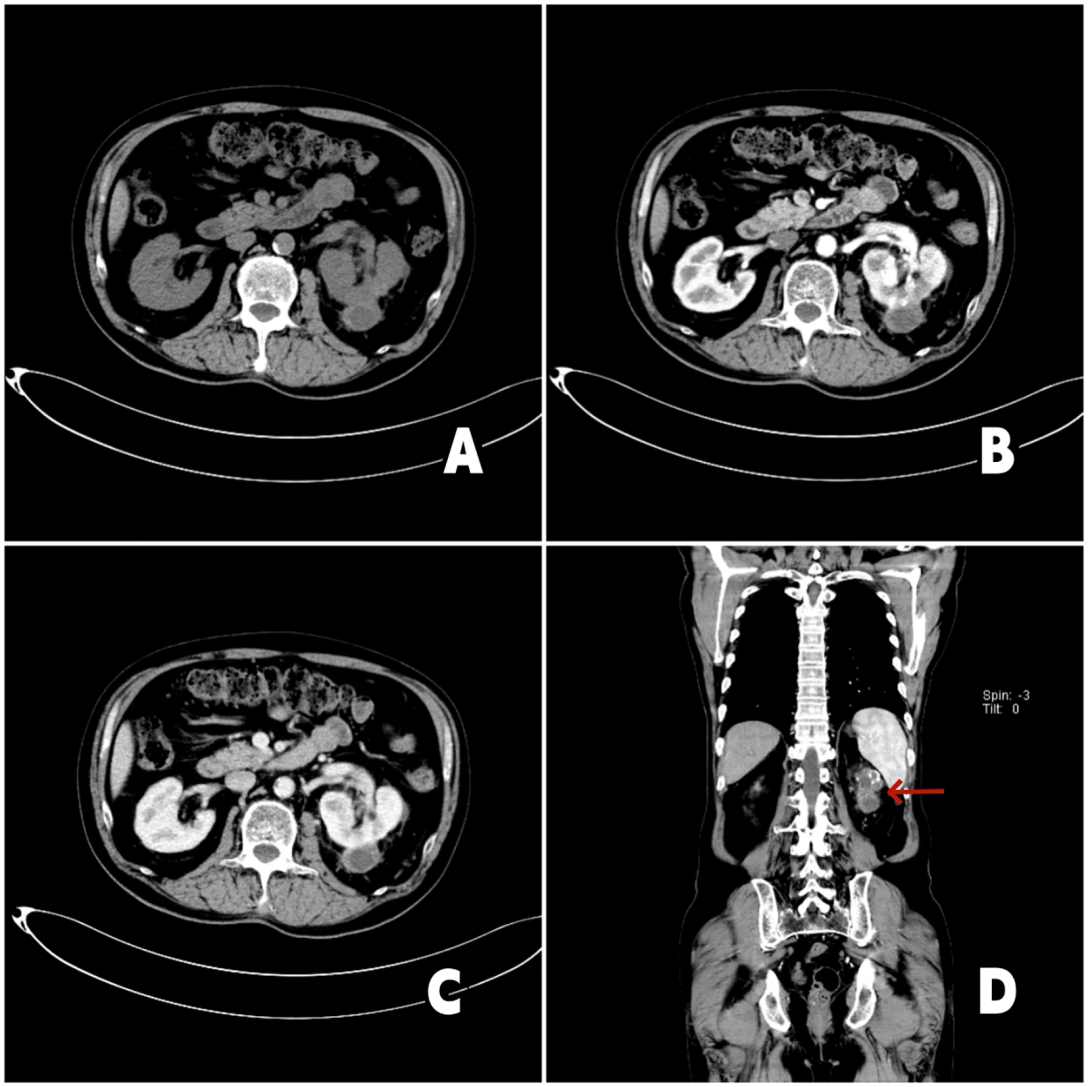
2023-10-19 chest enhanced CT and urinary CT: **(A)** Axial CT imaging demonstrates reduction of the left renal tumor. **(B)** Arterial-phase contrast-enhanced CT imaging reveals irregular enhancement of the left renal mass. **(C)** The tumor thrombus within the left renal vein shows regression, measuring approximately 2.4 cm in length. **(D)** Coronal contrast-enhanced CT imaging (red arrow denotes the left renal tumor) shows that the left renal mass has decreased in size compared to prior imaging.

A 65-year-old male presented on March 10, 2023, with an incidentally detected left renal mass (maximum diameter: 8.8 cm) on Computed Tomography Urography (CTU) imaging, without hematuria, lumps or lumbago. Initial imaging (**Figure 1**) revealed a left renal tumor with invasion into the renal pelvis and calyces, accompanied by a left renal vein tumor thrombus (length: 2.9 cm), along with suspected metastatic lesions in the liver, bilateral lungs, mediastinal lymph nodes, and bilateral adrenal glands. His medical history included diabetes mellitus and hypertension. The patient refused the puncture biopsy. After a multidisciplinary discussion, immunotherapy with Toripalimab (240 mg every 3 weeks) was initiated on March 17, 2023. First follow-up imaging (April 29, 2023) demonstrated partial response: the left renal mass, pulmonary nodules, and mediastinal lymph nodes had decreased in size, with complete resolution of hepatic metastases. Immunotherapy was continued through June 29, 2023 (cycles 3-6). To enhance efficacy, pazopanib (800 mg daily) was added on June 30, 2023. However, treatment was interrupted after one month due to pazopanib-induced grade 3 hepatotoxicity, which resolved following hepatoprotective therapy. After a 2-month treatment hiatus, follow-up imaging (**Figure 2**) on October 9, 2023, revealed sustained tumor regression: the left renal mass had decreased to 4.6 cm (from 8.8 cm), the renal vein thrombus to 2.4 cm, and pulmonary metastases to 0.7 cm (from 1.4 cm). Hepatic and mediastinal lymph node metastases remained absent, while bilateral adrenal nodules were deemed likely benign adenomas. Notably, serum tumor markers (CEA, ferritin) were elevated, though imaging confirmed overall treatment efficacy.

**Figure 3:**
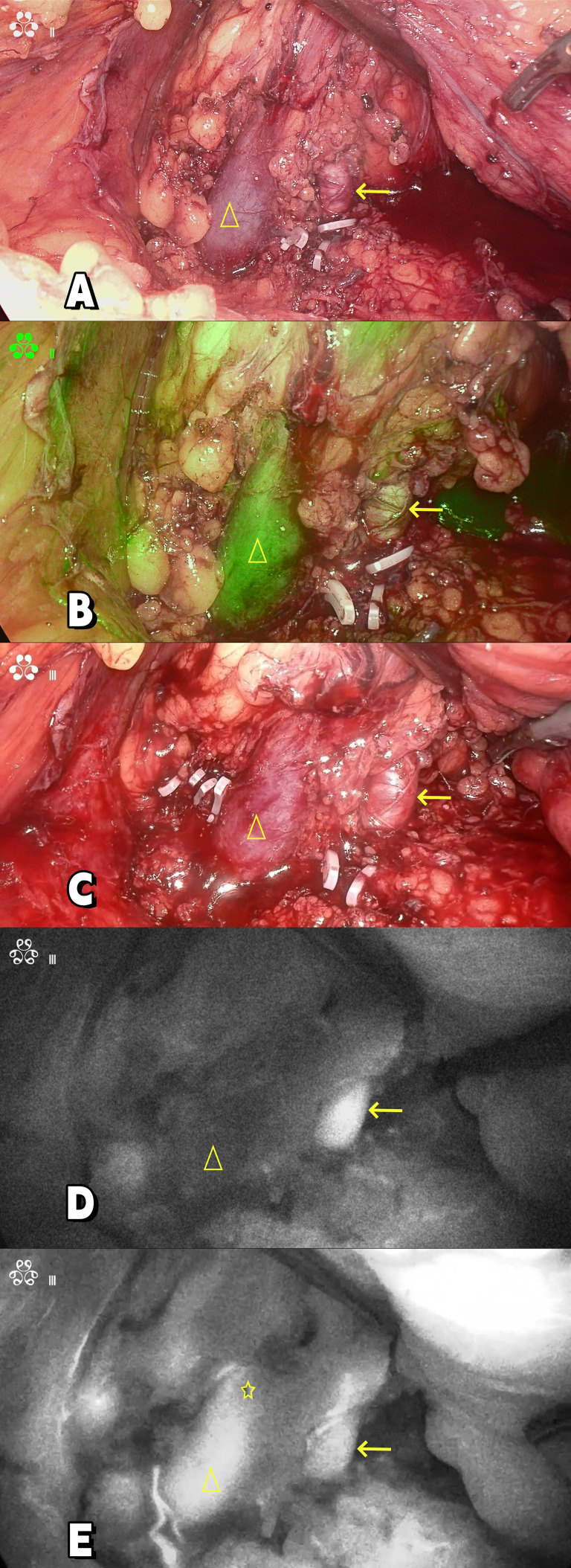
**(A, C)** Intraoperative image; **(B)** Composite fluorescence; **(D, E)** Near infrared fluorescence.“→”: Renal artery; “△”: Renal vein; ”☆”: Renal venous tumor thrombus.

**Figure 4:**
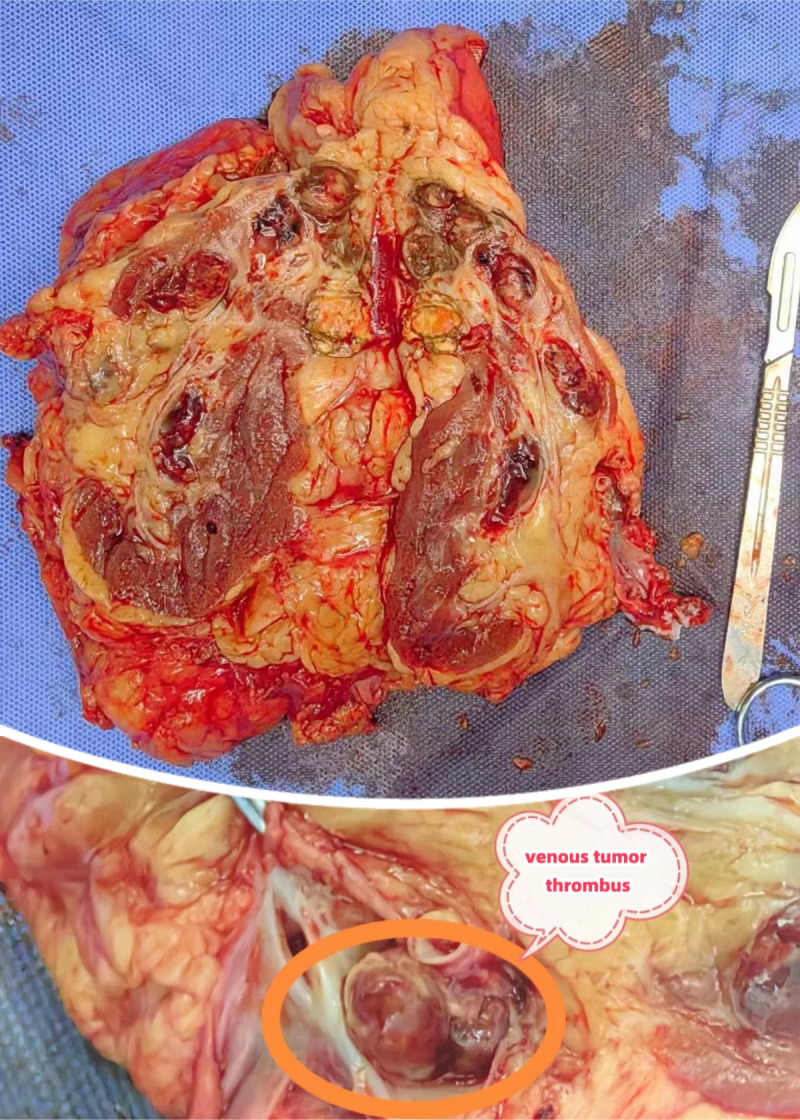
Surgical specimen Image.

After evaluation, the patient was in good physical condition, without systemic symptoms. Surgical resection of the primary left renal lesion could reduce most of the overall tumor burden. Moreover, the patient’s Memorial Sloan Kettering Cancer Center(MSKCC) risk score and International Metastatic Renal Cell Carcinoma Database Consortium (IMDC) risk score were both low to moderate risk, and there were surgical indications for cytoreductive nephrectomy(CN) for metastatic renal cell carcinoma. And the patient and his family had the willingness to undergo surgery. Therefore, our department performed a cytoreductive nephrectomy and thrombectomy(CNT) for metastatic renal cell carcinoma(mRCC) with renal vein tumor thrombus under the assistance of ICG fluorescence real-time imaging technology via the transabdominal approach laparoscopy under general anesthesia on November 7, 2023. The surgical position was the right oblique supine position (70°). During the operation, a four-hole operation channel was established. After fully dissociating and exposing the left renal vein and left renal artery, a total of 50 mg of ICG (12.5 mg per administration; Dandong Medical Creation, Dandong, China) was administered intravenously in divided doses to assess the left renal vein and tumor thrombus. After injecting ICG for 5–10 seconds during surgery, the fluorescence laparoscope (Nanjing Nuoyuan Fluorescence Lens, Nanjing, China) was set to detection of the luminescent ICG. The fluorescence laparoscope was alternated between white light, fluorescence, and black and white light, to observe and record the imaging characteristics of left renal artery and vein. First, in the near-infrared Fluorescence mode, the renal artery was visualized, and then the renal vein was visualized. When the renal vein was visualized, “filling defect” was found at the renal vein, thereby confirming the location of the left renal vein tumor thrombus (**Figure 3**). Finally the kidney was separated, and the kidney along with the adrenal gland, surrounding fat, and perirenal fascia was removed as a whole. The incision was enlarged to remove the specimen. The operation was successful. The operation time was 350 minutes and the blood loss was 400 ml. When dissected, the tumor thrombus in the renal vein was intact (**Figure 4**).

Histopathology confirmed clear cell renal cell carcinoma (WHO/ISUP grade 1, pT3aN0M1) with negative surgical margins and a left adrenal cortical adenoma. Postoperative recovery was uneventful.

Due to prior pazopanib intolerance, adjuvant therapy comprised sunitinib (3 months) and toripalimab (4 cycles). As of the latest follow-up (November 10, 2024), surveillance imaging showed no local recurrence or new metastases, with stable residual pulmonary nodules, consistent with durable disease control.

## 3 Discussion

Metastatic renal cell carcinoma is primarily managed with systemic drug therapy, complemented by local treatments like palliative surgery or radiotherapy for the primary or metastatic sites. Treatment selection hinges on a comprehensive assessment of primary and metastatic lesion characteristics, tumor risk stratification, and the patient’s performance status to devise an optimal combination treatment strategy (6).

Firstly, combined VEGFR targeted therapy and PD-1 monoclonal antibodies show promise in advanced renal cell carcinoma. Notably, studies highlight favorable outcomes with Toripalimab combined with targeted drugs in advanced cases, underscoring the strategy’s potential (7).

Secondly, CN combined with targeted drugs is still an optional treatment plan according to the results of the CARMENA study and several retrospective studies (8–10), at least for the following five situations: (1) Good physical condition (Eastern Cooperative Oncology Group[ECOG] score<2); (2) No systemic symptoms or mild symptoms; (3) Low metastatic burden or the surgery can reduce most of the overall tumor burden; (4) Medium and low-risk patients according to MSKCC or IMDC; (5) Severe local symptoms caused by renal tumors. Beyond that, radiotherapy is a possible alternative to surgery in patients who are not candidates for surgical intervention (11).

In our case, the patient benefited from preoperative targeted therapy and immunotherapy. The primary lesion shrank and the liver metastases disappeared. As a result, the patient was offered the opportunity for surgery. However, the patient’s renal vein tumor thrombus remained a major surgical challenge. 4%-10% of renal cell carcinomas are accompanied by venous tumor thrombus (3). The aggressive surgical resection of the affected kidney and tumor thrombus as the standard strategy for treating patients with renal cell carcinoma with venous tumor thrombus has been widely accepted (12, 13) and can bring survival benefits to patients (14). A recent meta-analysis found that TNM stage, Fuhrman grade, tumor necrosis, and the height of the tumor thrombus were significantly related to the postoperative survival of patients (15).

The surgical methods for renal cell carcinoma with tumor thrombus mainly include traditional open surgery, laparoscopic surgery, and robotic-assisted surgical techniques (16). Traditional surgery is associated with significant trauma and slow recovery. Robotic-assisted laparoscopic surgery on the other hand, has high equipment costs, making it difficult to promote in primary-level hospitals. Laparoscopic surgery can be carried out in most municipal or county-level hospitals in China. However, it still poses great challenges, especially when surgeons lack experience.

The key challenges during the operation include the difficulty in accurately determining the location of the renal vein tumor thrombus, the risk of inadequate resection length of the renal vein, which may lead to residual tumor thrombus, and the potential for tumor thrombus detachment during the procedure, thereby causing pulmonary artery embolism.

To enhance surgical efficiency and precision, and particularly to assist young surgeons in more accurately identifying blood vessels and tumor thrombi, the utilization of intraoperative visualization tools is imperative. Currently, the following are several commonly employed intraoperative vascular visualization tools:

Laparoscopic ultrasound is a technology that combines an ultrasound probe with a laparoscope. It can be inserted into the abdominal cavity through the working channel of the laparoscope to perform real-time imaging of blood vessels, assisting doctors in identifying blood vessels and avoiding surgical injuries. A study have shown that laparoscopic ultrasound can significantly reduce the operating durations in patients undergoing laparoscopic nephrectomy (17). In another preliminary study, laparoscopic radical nephrectomy with intraoperative, real-time ultrasonography can be satisfactorily applied to renal mass with a level 1 renal vein thrombus (18). However, this technology has limitations. It has restricted imaging range and depth, its image interpretation depends on professional experience, it is vulnerable to interference from gas in the abdominal cavity, and an ultrasound probe needs to be inserted during the operation.

Angiography equipment, such as Digital Subtraction Angiography (DSA), can clearly display the morphology, course, and lesions of blood vessels in complex laparoscopic surgeries by injecting contrast agents and using imaging techniques. However, its operation is complex and risky. The contrast agent may cause allergic reactions, and it requires highly skilled operators. The equipment and environmental requirements are stringent, and it is expensive, which limits its application scope. It also increases the surgical cost and preparation difficulty. At the same time, it prolongs the operation time and increases the risk of radiation exposure for patients and medical staff.

In contrast, ICG fluorescence laparoscopy can significantly improve this situation. The Indocyanine Green (ICG) Fluorescence Imaging System harnesses the unique properties of ICG, a near-infrared fluorescent dye. Upon intravenous injection, ICG binds to plasma proteins and circulates throughout the body (19). When excited by 785 nm excitation light, ICG emits near-infrared fluorescence at 815 nm. The camera system captures this fluorescence, enabling the real-time display of high-definition white light, fluorescence, and multimodal images on the screen. This technology facilitates the observation of blood perfusion, tracing of lymph nodes, and localization of tumor tissues.

In the context of laparoscopic surgeries, the system is particularly valuable for clearly visualizing the outline of blood vessels and blood flow dynamics, assisting surgeons in differentiating between various vascular structures. It plays a crucial role in identifying important arteries, veins, and tumor boundaries, thereby enhancing the precision of surgical procedures and minimizing vascular injuries and bleeding. In the field of urologic surgery, ICG has numerous applications (5), such as Partial Nephrectomy, Donor Nephrectomy, Kidney Transplantation, Radical Prostatectomy, Radical Cystectomy, Penile Cancer, Ureteral Surgery, Adrenalectomy and Varicocelectomy. Chul Jang Kim et al. (20) reported a case about the application of the ICG fluorescence imaging method in laparoscopic resection of a solitary retroperitoneal metastasis of renal cell carcinoma. Their report confirmed that ICG fluorescence imaging, with its high-sensitivity and specificity, can be a highly useful method for identification of metastatic small lesions of RCC during laparoscopic surgery. Jun Feng et al. (21) reported the clinical application of ICG fluorescence imaging navigation in pediatric renal cancer. It has been confirmed through their research that ICG fluorescence imaging is safe and feasible for renal cancers in children. In these reports, we have not seen any reports of its application in renal cell carcinoma with tumor thrombus. But Andrea Pansa et al. (22) reported a case which used ICG fluorescence guided anatomical segmentectomy for Hepatocellular Carcinoma(HCC) with portal thrombosis. This provides a certain theoretical basis for applying the fluorescence navigation technology of ICG to cases of renal cancer with tumor thrombus.

In our case, through intravenous injection of ICG, the real-time ICG fluorescence imaging technology can accurately map the filling defect of the renal vein, providing essential information for determining the location and boundaries of “tumor thrombi”. In the surgery for renal cancer with renal vein tumor thrombus, it has the following functions:①Real-time imaging of blood vessels, reducing the amount of surgical blood loss and improving surgical safety (23); ②Tumor thrombus localization; ③Shortening the operation time (24); Accurate navigation can reduce the exploration and trial-and-error process of doctors during the operation, thereby improving the surgical efficiency, shortening the operation time, and reducing the trauma and anesthesia risks of patients.

Moreover, indocyanine green (ICG) is relatively inexpensive. A 25-mg vial costs only approximately $14.2, making it highly suitable for promotion in primary-level hospitals.

Given the small number of cases of ICG-guided indocyanine green fluorescent laparoscopic CNT for metastatic renal cancer with renal vein tumor thrombus currently carried out by us, more cases are needed for further verification in the future.

## 4 Conclusion

In this preliminary study, Indocyanine Green (ICG) fluorescence-guided laparoscopy demonstrates satisfactory applicability in treating renal cell carcinoma with renal vein thrombus. The ICG fluorescence real-time imaging technology is noted for its real-time dynamics, convenience, and safety. Nevertheless, longer-term follow-up studies are crucial for a comprehensive evaluation of the role of ICG fluorescence-guided laparoscopy in urologic oncologic surgery.

## Data Availability

The original contributions presented in the study are included in the article/**Supplementary Material**. Further inquiries can be directed to the corresponding author/s.
